# Physiological aspects of sex differences and Haldane’s rule in *Rumex hastatulus*

**DOI:** 10.1038/s41598-022-15219-1

**Published:** 2022-07-01

**Authors:** Andrzej J. Joachimiak, Marta Libik-Konieczny, Tomasz Wójtowicz, Elwira Sliwinska, Aleksandra Grabowska-Joachimiak

**Affiliations:** 1 Department of Plant Cytology and Embryology, Faculty of Biology, Institute of Botany, Jagiellonian University, Gronostajowa 9, 30-387 Kraków, Poland; 2 Franciszek Górski Institute of Plant Physiology, Polish Academy of Sciences, Niezapominajek 21, 30-239 Kraków, Poland; 3 Department of Plant Breeding, Physiology and Seed Science, Faculty of Agriculture and Economics, University of Agriculture in Krakow, Łobzowska 24, 31-140 Kraków, Poland; 4 Laboratory of Molecular Biology and Cytometry, Department of Agricultural Biotechnology, Bydgoszcz University of Science and Technology, Kaliskiego Ave. 7, 85-789 Bydgoszcz, Poland

**Keywords:** Evolution, Plant sciences

## Abstract

Haldane’s rule (HR, impairment of fertility and/or viability of interracial hybrids) seems to be one of few generalizations in evolutionary biology. The validity of HR has been confirmed in animals, and more recently in some dioecious plants (*Silene* and *Rumex*). Dioecious *Rumex hastatulus* has two races differing in the sex chromosome system: Texas (T) and North Carolina (NC), and T × NC males showed both reduced pollen fertility and rarity—two classical symptoms of Haldane’s rule (HR). The reduced fertility of these plants has a simple mechanistic explanation, but the reason for their rarity was not elucidated. Here, we measured selected physiological parameters related to the antioxidant defense system in parental races and reciprocal hybrids of *R. hastatulus*. We showed that the X-autosome configurations, as well as asymmetries associated with Y chromosomes and cytoplasm, could modulate this system in hybrids. The levels and quantitative patterns of the measured parameters distinguish the T × NC hybrid from the other analyzed forms. Our observations suggest that the rarity of T × NC males is caused postzygotically and most likely related to the higher level of oxidative stress induced by the chromosomal incompatibilities. It is the first report on the physiological aspects of HR in plants.

## Introduction

A longstanding observation showed that in F1 hybrids between animal races or species, heterozygous sex is often impaired in terms of fertility and/or viability. This principle is commonly known as Haldane’s rule (HR) and seems to be one of few generalizations in evolutionary biology, or even the only regularity of speciation^1–3^. The validity of HR has been confirmed in gonochoric animals showing different, independently originated sex chomosome systems^4,5^. It has been shown that male sterility is about 10 times more prevalent than male inviability in hybrids^6^ and that the strength of isolation barriers differs significantly between reciprocal crosses^7^. These observations suggest that the HR-related reproductive isolation is most probably a two-step process and requires uniparentally inherited factors.

In the classical sense, HR refers only to the relationship between heterogamety and the sex of hybrids suffering the greatest fitness loss in animals, manifested as infertility and/or rarity of this sex^1^. Although the cause of HR proved to be more complex than previously thought^8–12^, it is related to sex chromosomes configuration and caused intrinsically, thus it can be observed even under optimal laboratory conditions^5,13^.

HR does not apply to the vast majority of angiosperm plants, as they are bisexual and have no sex chromosomes. Recently, it was revealed that HR in its canonical form can be extended to dioecious plants having X/Y sex chromosomes^14^. So far, however, there have been only three reports on this subject, and they concern only hybrids between plants differing in sex chromosome systems^14–16^.

As regards the X/Y sex chromosomes, three most supported theories (the dominance theory, faster X theory, and faster male theory) underline a special role of X-linked incompatibilities in HR^9,11,17,18^. This disproportionately high influence of X chromosome on the fertility and viability of interracial hybrids is commonly known as the “large X-effect”. Most probably, it is facilitated by the hemizygosity and faster divergence of X chromosome^19^. It can be expected that the degree of the Y chromosome degeneration and the X chromosome size should influence this effect, because they determine the number of X-linked genes being exposed in the heterogametic sex. In some cases, such as in *R. hastatulus*, poor fertility of hybrid males may result from the incorrect chromosome distribution in meiosis^16^, but inviability is difficult to explain without considering postzygotic epistatic interactions between discordant gene loci (Dobzhansky–Muller incompatibilities, DMIs)^20^. Haldane suggested that they resulted from the X–Y incompatibilities, whereas modern DMI-based models underline the complex interactions between X-linked and autosomal loci^5,8,11^.

The asymmetries in the viability of reciprocal hybrids observed in various taxa may also indicate cyto-nuclear incompatibilities^17^, but for male-heterogametic organisms possessing well-differentiated sex chromosomes and showing a large X-effect they were only rarely considered. It was because the conflict between the X chromosome and organellar genomes seems impossible due to the fact that in male hybrids the X chromosome and cytoplasm come from the same race. On the other hand, the cyto-nuclear incompatibility involving the X chromosome may appear in hybrid females and contribute to a decrease in their viability^8^. In ZZ/ZW taxa the described relationships are reversed and the cytonuclear conflict works in line with HR, impairing heterogametic sex, i.e. females. For instance, incompatibilities between Z-linked and mitochondrial genes are considered the main cause of HR and postmating reproductive isolation in birds^21^. The role of the cyto-nuclear incompatibilities in hybrid breakdown is relatively well understood, but mainly in interspecific hybrids of hermaphroditic plants. It often manifests as cytoplasmic male sterility (CMS), induced by incompatibility between cytoplasmic alleles (mitochondrial in most cases) from one parent and nuclear alleles from the other parent^22,23^. In many cases CMS showed asymmetry in reproductive isolation, i.e. differences between reciprocal crosses, which may determine the direction of gene flow between diverging populations in nature^7,24^. Much rarer are plastome-genome incompatibilities (PGI). They often impair PSII activity and produce bleached/variegated phenotypes in hybrids^23^. In *Oenothera* this type of incompatibility is the major factor causing hybrid breakdown and maintaining reproductive barriers between species^25^.

The majority of DMI-based theories refer almost exclusively to incompatibilities between genes. However, these disturbances must have certain physiological consequences, most probably related to the loss or impairment of functioning of enzymatic proteins (especially homomeric ones), and/or dysfunction of the organelles involved in energy production. The organellar protein complexes are encoded by nuclear and organellar genes, leading to genetic interactions between the nucleus and the organelles. Therefore, coordination between organellar and nuclear genomes is crucial for proper functioning of plant cells. The disruption of the genetic cytonuclear cooperation may lead to metabolic changes and overproduction of reactive oxygen species (ROS) and harmful oxidative stresses^26,27^. This in turn triggers induction of the complex response of the antioxidant system consisting of—among others—enzymatic antioxidants, such as superoxide dismutase (SOD) and catalase (CAT). These homomeric enzymes, like most of the proteins necessary for functioning of the organelles, are encoded in the nucleus, thus nuclear DMIs play a significant role in these phenomena. Such physiological responses are a consequence of hybridity in general and were observed in a wide range of organisms^21,23,26–29^. In animals, the main source of ROS are mitochondria, but in plants, large amounts of reactive oxygen species are also produced in chloroplasts. Within them, photosystem II (PSII) is particularly susceptible to oxidative damage^30^.

Perhaps the most important in HR is the combination of heterogamety and hybridity. Although the genetic differences (between sexes, between hybrids and parents) are crucial, the lowered fitness of hybrid genotypes must be related to its physiological specifity. Explaining of this specifity is important to understand why some genotypes perform worse than others. Unfortunately, most of the current physiological plant research on this topic focused on genders in dioecious plants without sex chromosomes or hermaphroditic hybrids subjected to different, mostly external biotic and abiotic stresses^31–37^. It was demonstrated that under stressful conditions the physiological differences between sexes, between parents and hybrids and between reciprocal hybrids may have a negative effect on the fitness of certain genotypes. According to Juvany and Munné-Bosch^38^, external stresses or their combination can even produce physiological differences between genotypes, not disclosed under favorable conditions. This does not make it easier to understand the physiological determinants of HR, because according to DMI models, the decisive role in this phenomenon is played by internal stresses related to interactions between discordant nuclear genes and to cyto-nuclear incompatibilities.

Basically, the mentioned patterns should apply to interracial hybrids in plants with heteromorphic sex chromosomes, although due to the lower level of the Y chromosome degeneration^39^, a different spectrum of HR causes can be expected, e.g., limitations of the large X-effect and a greater role of Y-autosome or X–Y incompatibilities. So far, there has hardly been any research on this subject. An important step towards clarifying this issue was the recent research on *Silene* conducted by Krasovec et al.^40^. Based on analysis of more than 1000 chromosomal loci, the authors found that 80% of X-linked genes had functional equivalents (gametologs) on the Y chromosome, and showed a lack of faster-evolving genes at hemizygous X-chromosome sites. This suggests that the two most important factors causing the large X-effect—the Y-degeneration and the faster X evolution—may be less effective in plants. On the other hand, such an effect could be supported by the relatively higher expression of X-linked alleles in males^41^. The available data on this subject, however, are not precise because increased expression of the Y gametologs was also reported^42^.

From about 20 angiosperm species with well-differentiated, heteromorphic sex chromosomes^43^, only American annual herb *Rumex hastatulus* possesses two chromosomal races differing in the sex chromosome system. The race Texas (T) has a simple XX/XY sex chromosome system and four pairs of autosomes, and the race North Carolina (NC) has a polymorphic XX/XY1Y2 sex chromosome system and three autosome pairs^44^. Basing on the satellite DNA divergence, the age of North Carolina race was estimated to be 600,000 years^45,46^. However, according to Beaudry et al.^47^, the neo-sex chromosomes of this race are probably much younger—about 180,000 years old. Smith^48^ hypothesized that the NC chromosome set had originated from the autosome-sex chromosome translocations in T race. Our in situ hybridization (FISH) studies fully supported the translocation hypothesis and showed which autosome pair was involved in chromosome rearrangements^49^.

Despite the differences in the karyotype structure, two *R. hastatulus* races hybridize in nature^50^. It was also possible to obtain interracial *R. hastatulus* hybrids experimentally^16^. This provided an opportunity to analyze the composition of the resulting chromosomal complexes, meiotic behavior of the chromosomes derived from different races, and fertility of F1 males. NC × T hybrids manifested an unbiased sex ratio, regular course of male meiosis (with the zig-zag segregating sex-trivalent) and high pollen viability, in which they did not differ from the parental races. On the other hand, T × NC males showed both reduced pollen fertility and rarity (M:F ratio = 1:1.7)—two classical expressions of HR. It was established that the reduced fertility of these males was a direct consequence of formation of two separate bivalents including sex chromosomes in meiosis. Due to independent segregation of their components, 25% of microspores and the resulting pollen grains had chromosomal deficiencies and could not develop properly^16^. However, the cause of the rarity of T × NC males has yet to be determined. It may be caused prezygotically, or postzygotically by regulatory disruption of physiological processes in the hybrid caused by DMIs. Both options deserve a detailed study, but the latter seems particularly interesting because little is known about the physiological differences between males and females in plants possessing sex chromosomes^32,38^, and there is absolutely no information on the physiological aspects of HR in these plants.

To address these questions we analyzed the proportion of male- and female-determining pollen grains in original *R. hastatulus* races and compared physiological parameters related to the photosynthetic activity and antioxidant defense system in these races and their reciprocal hybrids (T, NC, T × NC and NC × T), paying special attention to the difference between sexes.

## Results

### Pollen genome size

**Figure 1:**
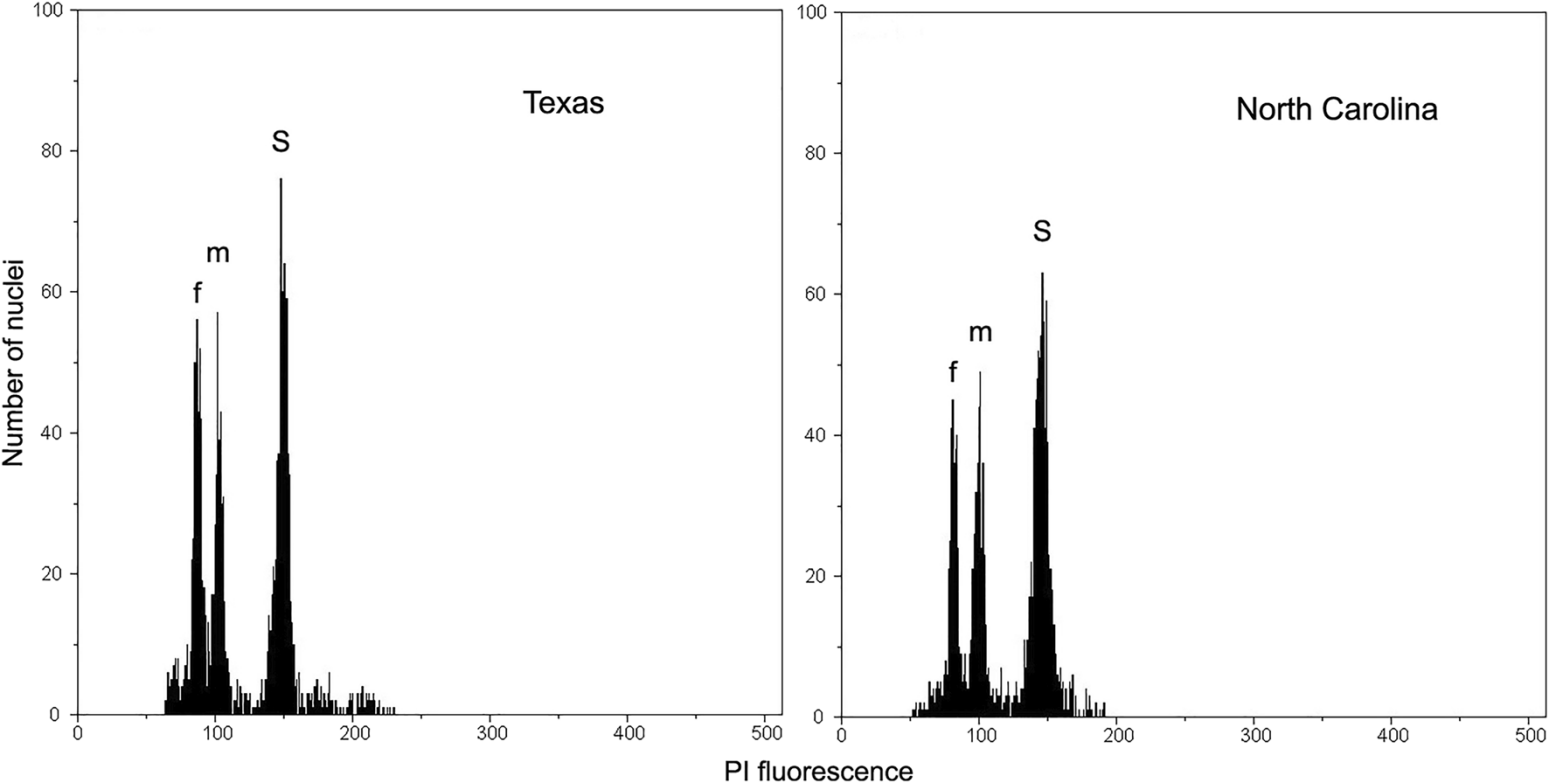
Selected FCM histograms of nuclear DNA content in PI-stained nuclei isolated from pollen grains of two races of *Rumex hastatulus*, Texas and North Carolina. *f* female nuclei, *m* male nuclei, *S* internal standard: *Petunia hybrida*, 2C = 2.85 pg.

FCM (flow cytometry) analysis enabled identification of peaks corresponding to female- and male-determining pollen nuclei in the parental races and measuring the male and female holoploid genome size (Fig. 1, Table 1). The difference between the size of single male and female genome was large enough to be cytometrically detected and similar in both races (18.6% in T vs. 17.2% in NC). The frequency of male- and female-determining pollen grains did not differ significantly from parity. The male parent of T × NC hybrid showed the proportion of sex-determining pollen close to 1:1.

**Table 1:** Flow cytometric measurements of DNA content and frequency of male- and female-determining pollen grains in two *R. hastatulus* races (*T* Texas, *NC* North Carolina).

Race	F_det_ [1C_F_, pg]	M_det_ [1C_M_, pg]	%M_det_	M_det_:F_det_
T	1247 [1.654 ± 0.024]	1013 [1.962 ± 0.027]	44.82	1:1.21
NC	1444 [1.618 ± 0.024]	1434 [1.897 ± 0.032]	49.83	1:1.01

*F*_*det*_ number of nuclei derived from female-determining pollen grains, *M*_*det*_ number of nuclei derived from male-determining pollen grains, *1C*_*F*_ female genome size, *1C*_*M*_ male genome size.

### Chlorophyll a fluorescence

The analyses of chlorophyll *a* fluorescence parameters showed that the tested plants did not differ much in this respect. Chlorophyll fluorescence parameters achieved from OJIP analysis may indicate electron fluxes between different components upstream, inside and downstream of PSII, thus they may indicate different points of stress-induced damages in photosystem II and photochemical reactions in photosynthesis. The only highly significant difference between plants of the same sex was found in the OJIP F0 (minimum Chl fluorescence yield in the dark-adapted state) values between T and T × NC females (p < 0.01). In photosynthetic samples, which have been kept in darkness, the electron acceptor side of PSII is mostly in the oxidized state (i.e., the PSII reaction centers are open, and the fluorescence intensity is minimal, F0). The increase in the minimum fluorescence yield of the dark-adapted sample (F0) can be caused by the lack of energy transfer from the antenna to the PSII reaction center.

With regard to basic measurable values (F0 and Fm-maximum chlorophyll fluorescence yield in the dark-adapted state) and psi_0 (electron transport flux in the electron transport chain), the difference between sexes in all analyzed forms was insignificant (p > 0.05 in each case). In T × NC hybrid, however, the Fv/Fm ratio (the ratio of variable to maximum fluorescence after dark-adaptation, represents maximum quantum yield of PSII, in most higher plants having usually the value in the range of 0.78–0.84) revealed a significant between-sexes difference (p = 0.0106), but this parameter was higher in males. Generally, most of the measured photochemical parameters were higher in males than in females (Table S1, Fig. S1). The detected differences seems to be not large enough to indicate the influence of internal stress on the course of photosynthesis. The absolute data of chlorophyll fluorescence (F0, Fm, Fv) might suggest differences in PSII structure in terms of antenna size in hybrids, whereas the relative parameters (Fv/Fm, psi0) calculated from absolute data indicate some impairment of PSII construction and photochemical reactions in hybrids but without negative influence on the total efficiency of photosynthesis.

### LMW antioxidant activity

**Figure 2:**
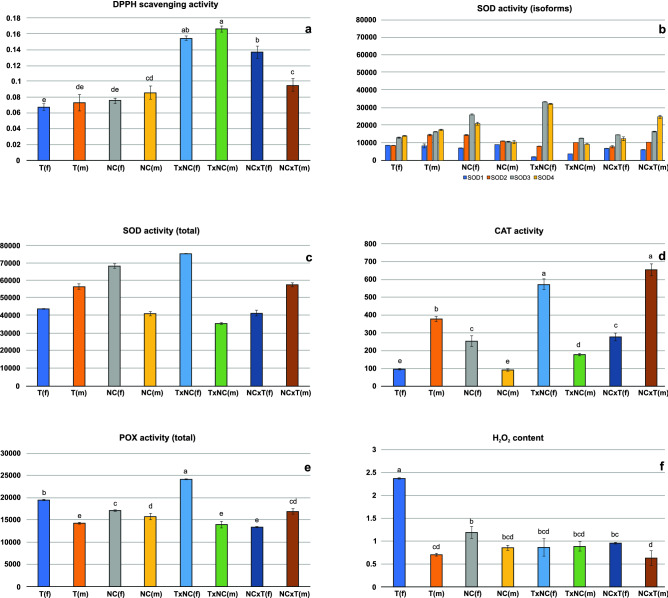
Analysis of selected physiological parameters in T, NC, T × NC and NC × T *R. hastatulus* forms. (f)—females, (m)—males; (**a**) DPPH free radical scavenging activity (in µmol dm^−3^ of Trolox equivalents); (**b**) activity of four detected SOD isoforms (a.u.); (**c**) total SOD activity (a.u.); (**d**) CAT activity (a.u.); (**e**) POX activity (a.u); (**f**) endogenous H_2_O_2_ content (in μM H_2_O_2_/g FW).

**Figure 3:**
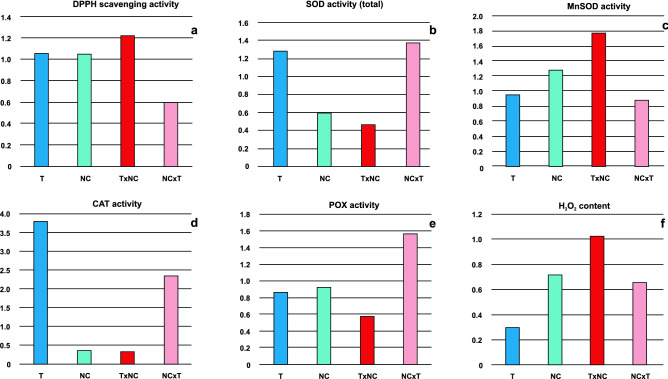
M/F ratios calculated for selected parameters of antioxidant system in four *R. hastatulus* forms. (**a**) DPPH free radical scavenging activity; (**b**) total SOD activity; (**c**) MnSOD activity; (**d**) CAT activity; (**e**) POX activity; (**f**) endogenous H_2_O_2_ content.

The genic incompatibilities can cause disturbances in plant metabolism leading to generation of oxidative stress by overproduction of reactive oxygen species (ROS). In order to avoid harmful effect of ROS on plant cell functioning the machinery of plant antioxidant system is activated. Our first approach was to assess the total antioxidant capacity of plant extracts by analysis of DPPH free radical scavenging activity. The total LMW antioxidant activity in the analyzed plants varied between 0.084 and 0.174 mg Trolox eqv. 100 mg^−1^ FW. It was established that in NC × T females and in both sexes of T × NC hybrid the LMW antioxidant activity was substantially higher than in their parents (Fig. 2a). In NC × T males, however, it did not differ considerably from that observed in North Carolina males (p = 0.069). There were highly significant differences between males and females in both hybrids (p < 0.001), with contrasting patterns with respect to quantitative relationships. In T × NC the substantially higher LMW activity was observed in males, whereas in NC × T in females. The between-sexes differences in the parental races were small and insignificant (p = 0.448 and p = 0.136, respectively). In all analyzed forms except NC × T, the measured values were lower in females. The M/F value peaked in T × NC males (Fig. 3a).

### SOD, CAT and POX activity

Furthermore, we analyzed the activity of three enzymes responsible for management of ROS homeostasis and ameliorating oxidative stresses—superoxide dismutase (SOD), catalase (CAT) and peroxidase (POX).

In all analyzed plants the activity of four SOD isoforms was detected: MnSOD (SOD1) and three CuZnSOD (SOD2-4) (Fig. S2). The activity levels of individual SOD isoforms and of total SOD activity do not show discernible patterns distinguishing hybrid plants (Fig. 2b,c). There were, however, significant differences (p < 0.001) between opposite sexes in all *R. hastatulus* forms. The males possessing the Y_T_ chromosome (Texas race and NC × T hybrid) manifested higher total SOD activity than females, whereas in males with neo-Y chromosomes (NC race and T × NC hybrid) this parameter was lower than in females. The T × NC hybrid showed the lowest M/F ratio regarding total SOD (Fig. 3b) and although males are clearly superior to females in terms of the activity of MnSOD (Fig. 3c), the contribution of this component to the total SOD activity was negligible.

The CAT activity also indicated considerable differences between opposite sexes (p < 0.001) in all analyzed forms. Much higher values were detected in T × NC females and NC × T males, whereas in T × NC males and NC × T females they were within the range observed in the parent races (Fig. S3, Fig. 2d). As with total SOD, males possessing Texas Y chromosome showed higher values than females, whereas males with neo-Y chromosomes had lower activity values than females (Fig. 3d).

In the analyzed plants the activity of four peroxidase forms was detected (Fig. S4). The activity of the POX1 isoform was observed in all plants, POX4 in both sexes of T race and in male NC × T hybrid, in which the activity of two additional isoforms of this enzyme was also registered (POX2 and POX3). Although the POX1 was by far the most active in all analyzed plants, the additional isoforms made a considerable contribution to the total peroxidase activity (12.1% in T females, 26.2% in T males, and 19.2% in NC × T males) (Table S2). Nevertheless, the highest total activity of this enzyme was recorded in T × NC females, in which only the common POX isoform was observed (Fig. 2e).

There were significant differences (p < 0.001) in the POX activity between opposite sexes in all analyzed forms. In two parental races and T × NC hybrid, males showed lower POX activity than females, whereas in NC × T hybrid the activity of this enzyme was almost 1.6 times higher in males (Fig. 3e).

### H_2_O_2_ amount

Since the activity of three analyzed enzymes influences the amount of endogenous hydrogen peroxide, we examined its level in the studied plants (Fig. 2f). Surprisingly, by far the highest H_2_O_2_ amount was observed in Texas females, whereas in hybrids (both males and females) it was much lower, in the range recorded in three other parental genotypes. In T × NC hybrid the H_2_O_2_ level was very similar in both sexes, and this form stood out from the other analyzed plants in terms of the M/F ratio (Fig. 3f). In other forms H_2_O_2_ amounts were higher in females and in the pair-wise comparisons the differences between sexes appeared to be statistically significant (p < 0.001 in T, p = 0.015 in NC, and p = 0.026 in NC × T).

### Statistical evaluation

To sum up, the share of variance components in shaping the analyzed parameters ranged from 93.8% (H_2_O_2_) to 99.8% (SOD3). For all analyzed parameters, race/gender had a decisive influence on their variability. T × NC males showed the lowest SOD and CAT activity among three hybrid genotypes with DPPH values pointing to elevated stress levels, which indicate that they may have problem with enzymes scavenging reactive oxygen species. On the other hand, the activity of these enzymes was high in females of this hybrid. In the second hybrid, SOD activity in both sexes was within the range observed in the parental forms, whereas CAT activity in males was more than twice that of females (and highest in all the plants tested).

We employed the orthogonal contrasts analysis to examine the effects of gender and cytoplasm on given parameters (Table 2). In general, higher values were recorded for females and plants possessing T cytoplasm. Interestingly, the inverse tendency was demonstrated for SOD1 and SOD2. There was no differentiation only between sexes in the effect on DPPH free radical scavenging activity and between cytoplasm in the effect on total SOD activity and CAT activity.

**Table 2:** The results of orthogonal contrast analysis in *R. hastatulus* plants; the values of nine parameters related to oxidative stresses were considered.

Contrast	F < > M	C_T_ < > C_NC_
DPPH	F = M (p = 0.226)	C_T_ > C_NC_ (p < 0.001)
CAT	F < M (p = 0,010)	C_T_ = C_NC_ (p = 0.173)
SOD-t	F > M (p < 0.001)	C_T_ = C_NC_ (p = 0.100)
SOD1	F < M (p = 0.001)	C_T_ < C_NC_ (p < 0.001)
SOD2	F < M (p < 0.001)	C_T_ < C_NC_ (p < 0.001)
SOD3	F > M (p < 0.001)	C_T_ > C_NC_ (p < 0.001)
SOD4	F > M (p < 0.001)	C_T_ > C_NC_ (p < 0.001)
POX-t	F > M (p < 0.001)	C_T_ > C_NC_ (p < 0.001)
H_2_O_2_	F > M (p < 0.001)	C_T_ > C_NC_ (p < 0.001)

*F* females, *M* males, *C*_*T*_ cytoplasm of Texas, *C*_*NC*_ cytoplasm of North Carolina, *F* <  > *M* females [T(f) + NC(f) + T × NC(f) + NC × T(f)] vs. males [T(m) + NC(m) + T × NC(m) + NC × T(m)], *C*_*T*_ <  > *C*_*NC*_ T cytoplasm [T(f) + T(m) + T × NC(f) + T × NC(m)] vs. NC cytoplasm [NC(f) + NC(m) + NC × T(f) + NC × T(m)].

**Figure 4:**
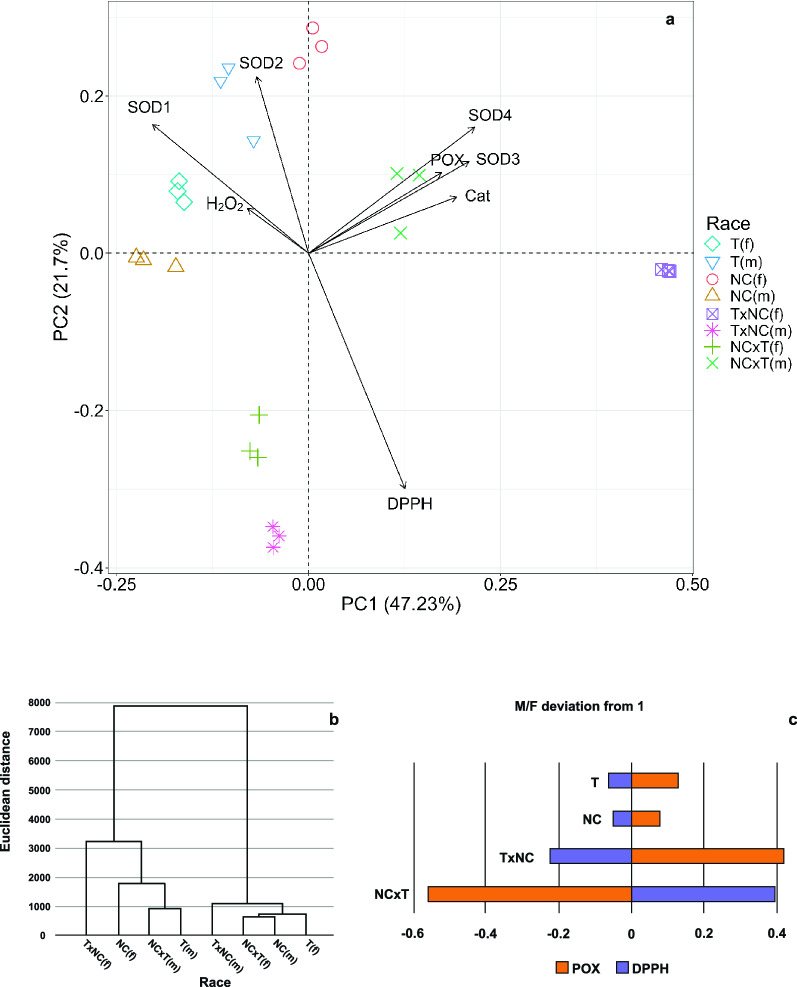
(**a**) Principal component analysis (PCA) biplot showing the relationship between the plant genotype and the activity of CAT, SOD, POX, free radical scavenging activity (DPPH) and the amount of H_2_O_2_; (**b**) dendrogram of *R. hastatulus* genotypes based on Ward’s hierarchical clustering; (**c**) deviation from M/F equilibrium in activity of POX and LMW antioxidants (DPPH).

To increase interpretability of the parameters related to the oxidative stress response, we performed the Principal Component Analysis (PCA) (Fig. 4a). Two highlighted components (PC1 and PC2) explained almost 69% of the total variance. The PC1 accounts for about 47% of variance. By far the greatest positive effect on this component was exerted by the activity of CAT, SOD3 and SOD4 isoforms, and POX. A small but positive effect on PC1 was exerted by antioxidant potential (DPPH). There was a positive correlation between catalase activity, activity of SOD3 and SOD4 isoforms, and POX activity. On the other hand, the antioxidant potential (DPPH) was not correlated with the activity of this group of enzymes. The greatest negative impact on PC1 was made by SOD1 (MnSOD) activity, which, along with SOD2 activity, was negatively correlated with the antioxidant potential (DPPH). The activity of the SOD1 isoform was significantly correlated with the content of H_2_O_2_. The second component (PC2) explains about 22% of the variability, with the activity of the SOD2 isoform having the greatest positive influence on it. The greatest negative influence on this component was detected in the case of the free radical scavenging activity (DPPH).

It can also be inferred from the PCA biplot that the NC × T males were characterized by higher catalase activity, peroxidase activity, and correlated SOD isoforms (hence, more efficient H_2_O_2_ reduction relative to the female individuals). On the other hand, Texas males and North Carolina females showed higher SOD2 activity. H_2_O_2_ content was the least determinant of variation between individuals (the shortest vector), which was confirmed using the Tukey test to compare the means. Plants showing the highest DPPH values were spaced from the remaining forms. The T × NC males were closer to NC × T females than to the T × NC females, which occupied the most distant position.

The Ward’s hierarchical analysis based on the original dataset showed two clearly distinct clusters composed of four plants each; a higher similarity between individuals was observed in the right one (Fig. 4b). The genders and forms were mixed up, and the only regularity was separation of the sexes belonging to a given form between these clusters. In the left cluster the greatest similarity was recorded for the NC × T and the T males (different cytoplasm, the same Y chromosome), in the right cluster—the NC × T females and NC males (same cytoplasm, different sex chromosomes). In each cluster, the T × NC hybrid occupied a separate position.

## Discussion

The DMI-based models assume a gradual accumulation of mutations on the rapidly evolving sex chromosomes and deepening of initially weak or even non-existent isolation barrier over time^18^. HR refers to the initial stage of this long-term process, so the intraspecific hybrids and young, differentiating/transitioning sex chromosome systems may be particularly useful in studying this phenomenon. Due to the above features and the complete lack of in-depth studies on HR in plants, interracial hybrids of *R. hastatulus* seemed to be a particularly promising research object.

In our previous experiment involving plants grown from F1 seeds obtained from free pollination in a vegetation room (with 1.5 m distance between male and female plants), only the T × NC hybrid manifested a significant (1:1.7) deviation from the balanced sex ratio^16^. In our opinion, under these conditions certation could not play an important role (or should similarly affect the offspring sex ratio in all analyzed forms). Thus, most likely, the reason for the rarity of T × NC males was different. The present FCM studies revealed that the proportion of male- and female-determining pollen grains was close to 1:1 in NC males, thus the rarity of T × NC males probably resulted from their lower viability.

To explain the internal factors that may affect the viability of *R. hastatulus* genotypes we compared selected physiological parameters in the parental races and their hybrids growing under optimal conditions. It was established that as far as chlorophyll *a* fluorescence parameters are concerned, they were similar in all the plants tested and the T × NC hybrid did not stand out in this respect. According to Foyer^30^, internal and external stresses induce decreases in Fv/Fm ratios, which in turn may be related to downregulation of PSII photosystem. In T × NC hybrid the between-sexes difference in Fv/Fm ratio was significant, but the value of this parameter was higher in males than in females. Generally, most of the measured photochemical parameters were higher in males. This contrasted with the parameters associated with antioxidant protective systems, which proved to be relevant and genotype-dependent. Most of them showed a significant correlation with sex and cytoplasm, indicating an important role of chromosome configuration and parental organelles in generating oxidative stress and/or shaping cellular responses to it. In general, higher parameter values were recorded for females and plants possessing T cytoplasm, but deviations from this pattern were also observed, especially in the activity of CAT, SOD1 (MnSOD) and SOD2 (one of the Cu/ZnSOD isozymes). The PCA analysis confirmed the distinct patterns of activity of SOD1 and SOD2 isozymes in the analyzed plants.

Superoxide dismutase (SOD) is an essential element of the antioxidant system in cells exposed to oxygen. Its multiple isoforms are the first line of defence against harmful oxidative stresses. They detoxify the superoxide radicals by its dismutation to H_2_O_2_, which can later be converted into H_2_O by other enzymes^51^. According to the metal cofactor, SODs are classified into three major groups, localized in different cell compartments: FeSODs (in plastids), MnSODs (in mitochondria, peroxisomes and cell wall), and Cu/ZnSODs (in cytosol, peroxisomes and plastids)^52^. The SOD activity in hermaphrodite hybrid plants is often higher than in their parents, and reciprocal hybrids may or may not exhibit asymmetry in this regard^37,53^. Determining what it is like in dioecious *R. hastatulus* is problematic due to the large differences between sexes in each analyzed form. A comparison of the mean values calculated for males and females together suggests that the total SOD activity in reciprocal *R. hastatulus* hybrids was between the parents’ values. On the other hand, T × NC females showed significantly higher, and T × NC males significantly lower total SOD activity than any other *R. hastatulus* plant.

It seems that the Y chromosomes have a strong influence on the SOD activity profiles: in cytoplasmatically different forms with Texas Y chromosome the total SOD activity was higher in males than in females, but in forms possessing the North Carolina neo-Y chromosomes, the SOD activity was much lower in males. There were, however, four SOD isoforms in the analyzed plants, and one of them, MnSOD (SOD1) showed the highest M/F ratio in T × NC plants. This may indicate mito-nuclear conflict involving the North Carolina Y chromosomes in this hybrid. Berwal et al.^53^ suggested that MnSOD did not follow the Mendelian pattern of inheritance, that is, reciprocal crosses showed a MnSOD isoform pattern similar to their mother plants. Such a relationship was not observed in *R. hastatulus*, where the MnSOD activity was similar in T, NC and NC × T and significantly lower in T × NC plants.

A similar to total SOD, Y-dependent pattern of M/F ratio was also observed for catalase, another enzyme responsible for the maintenance of redox homeostasis. Catalase transforms two H_2_O_2_ molecules into H_2_O and O_2_, and in this reaction no additional reductant is required^54^. According to Mhamdi et al.^55^, catalases may be up-regulated or down-regulated under various stresses and no general pattern of changes in the catalase activity in response to stress was observed. The activity of this enzyme varied between *R. hastatulus* genotypes and was generally lower in parents than in hybrids. Definitely the highest values of this parameter were recorded in T × NC females and NC × T males. Among all analyzed plants, the T × NC hybrid showed the lowest, and the NC × T the highest M/F ratio, for both CAT and total SOD activity.

The harmful excess of H_2_O_2_ could also be scavenged by peroxidases (POXs), a diverse group of enzymes differing in reducing cofactors. In contrast to catalases, peroxidases reduce H_2_O_2_ finally to water at the expense of other reductants, without generating O_2_^56,57^. Peroxidases, like SODs and catalases, are encoded by nuclear genes^58^. Enhanced expression of peroxidases has been shown to increase stress tolerance in plants^59^. Total POX activity was especially high in T and T × NC females and NC × T males. Only POX1 isoform was common to all genotypes, and it showed the highest activity in all analyzed plants. However, additional POXs exerted a non-negligible effect on the total peroxidase activity. POX4 was observed in both sexes of Texas race and NC × T males, whereas POX2 and POX3 only in NC × T males. The pattern of POX4 activity indicated that it is genotype-dependent and most probably associated with the Texas autosomes. However, the presence of its gametologs on both Texas sex chromosomes cannot be rejected. The activity of two other isoforms (POX2 and POX3) was revealed only in NC × T males, so it is difficult to determine their parental origin. Most likely, the activity of the additional POX isoforms is regulated so that the total peroxidase activity is sufficient to neutralize excess of hydrogen peroxide. Interestingly, the POX activity showed an inverse relationship to DPPH scavenging activity with respect to sex as evidenced by the calculated M/F ratios; if one of these parameters deviates negatively from equilibrium, the other deviates positively. Moreover, M/F deviations are clearly higher in hybrids than in parents (Fig. 4c).

The endogenous content of hydrogen peroxide was similar in the majority of the analyzed plants, and in most cases it was below 1 μM H_2_O_2_/g FW. Only in females of the original races it exceeded this value—in Texas almost 2.5 times. All these values are within the limits well tolerated by plants^60^ and may be related to specific requirements of a given form. H_2_O_2_ is a signaling molecule, and modulation of its concentration may be important in the control of nuclear gene expression and proper regulation of many physiological processes^30,60^. In *R. hastatulus*, requirements in this regard seem to be gender-specific, greater in females than in males. Only in T × NC hybrid, the concentration of H_2_O_2_ is equal in two sexes, which may indicate that males have trouble in maintaining this parameter on an appropriate level. The imbalance between the production and neutralization of ROS is thought to be the main cause of the harmful effects of oxidative stress^30,61^. It is difficult to determine whether the deviation from the gender-specific pattern of H_2_O_2_ concentration in T × NC hybrid is an indicator of such an imbalance in male plants, but it seems likely. One of the reasons of such imbalance may be the relatively low (compared to the opposite sex) level of CAT and POX activity in this hybrid.

The activity patterns of analyzed enzymes in hybrids showed no relationship with the particular X-autosome configuration. However, the influence of X-autosome configuration on the activity of LMW antioxidants was detected in DPPH analysis. Three hybrid forms possessing Texas X chromosome (both sexes of T × NC hybrid and NC × T females) revealed significantly higher DPPH scavenging activity than any other plant. They differed in cytoplasm, and the only common nuclear configuration that can be a source of genetic conflict in these plants was the co-occurrence of Texas X chromosome with the North Carolina autosome set. This chromosome combination was absent in hybrids showing significantly lower LMW antioxidant activity, i.e., in NC × T males (Table 3).

**Table 3:** Chromosome configuration and cytoplasm in reciprocal *R. hastatulus* hybrids.

Genotype	Chromosome configuration	Cytoplasm
T × NC (f)	X_T_·X_NC_·A_T_·A_NC_	C_T_
T × NC (m)	X_T_·Y_NC_·A_T_·A_NC_	C_T_
NC × T (f)	X_T_·X_NC_·A_T_·A_NC_	C_NC_
NC × T (m)	X_NC_·Y_T_·A_T_·A_NC_	C_NC_

*X*_*T*_*, Y*_*T*_ Texas sex chromosomes, *X*_*NC*_*, Y*_*NC*_ North Carolina sex chromosomes, *A*_*T*_ Texas autosome set, *A*_*NC*_ North Carolina autosome set, *C*_*T*_ Texas cytoplasm, *C*_*NC*_ North Carolina cytoplasm.

This may mean that three hybrid plants are subject to internal stress due to X-autosome incompatibility. Since only T × NC males showed reduced viability, it can be assumed that they were less able to handle this stress than female hybrids. Due to the complexity of the antioxidant system, consisting of enzymatic and non-enzymatic components^59^, it is difficult to pinpoint the reason for this, especially because the hybrid females differ significantly in the level of SOD, CAT and POX activity. Although three hybrid plants exhibit similarly high DPPH scavenging activity, males and females may differ in the composition of the LMW antioxidants, which may affect their antioxidant efficiency. For instance, in *R. thyrsiflorus*, another dioecious *Rumex* species with sex chromosomes, females showed three times higher content of catechins than males^62^.

The special role of sex chromosomes in HR is mainly due to the fact that they lose their ability to recombine and become specialized to enhance either male or female fitness. The process of their genetic differentiation depends to a large extent on inversions, which are the main causes of homology loss and subsequent degeneration of the Y(Z) chromosome resulting in hemizygosity of the sex chromosomes in heterogametic sex^63^. However, the sex chromosome system formed in a given taxon/species group is not always permanent. The occasional autosome-sex chromosome translocations can initiate dramatic changes to it, because they change the number of autosomes and sex chromosomes, affect the linkage and expression of many genes and alter the recombination patterns^64^. A wide range of such changes related to the transformation of the sex chromosome system was also observed in *R. hastatulus*^65,66^. All changes of this kind increase the genomic mismatches in hybrids between plants differing in the sex chromosome system. It is noteworthy that the only cases of HR in plants were reported in this type of hybrids^15,16^.

Another factor that may influence the antioxidant response may be cytoplasm. The orthogonal contrast analysis suggested the role of this factor in shaping the analyzed parameters, but its role can be clearly demonstrated only in plants with the same nuclear make-up and different cytoplasm, i.e., in females of reciprocal hybrids. The quantitative ratios calculated for each parameter showed distinct differences between the compared females (Table 4) in the activity of the antioxidant enzymes (except for SOD2). On the other hand, cyto-nuclear make-up seems to have no major effect on the H_2_O_2_ content and the activity of non-enzymatic antioxidants (DPPH) in these plants.

**Table 4:** Differences between hybrid females in selected parameters of the antioxidant system.

	DPPH	SOD-t	SOD1	SOD2	SOD3	SOD4	CAT-t	POX	H_2_O_2_
A/B	0.87	1.82	0.30	1.03	2.28	2.59	2.06	1.57	0.90

*A* T × NC(f), *B* NC × T(f), *SOD-t* total SOD, *CAT-t* total CAT.

The two key factors in Haldane’s rule in general (and in reduced viability in particular) are hybridity and sex affiliation. They determine the genetic difference between the affected organism and its closest relatives and—ultimately—the physiological properties responsible for HR. However, each of these factors can also act independently, which is particularly evident in plants. In hermaphroditic plants the physiological differences between hybrids and parents can play an important role in both homoploid and heteroploid speciation. In some cases, the hybridity can generate physiological novelties that increase plasticity and survival of newly established allopolyploids. Most of them arise from transposon activation, epigenetic changes inducing non-additive expression patterns and modifications in gene regulation^33,67–70^. It is generally believed that the primary cause of these changes is genome mismatch (“genomic shock”). Mostly, however, hybrid plants showed rather reduced fertility and/or viability, often resulting from cyto-nuclear incompatibilities^23^. Similar phenomena are observed in animals in which the reduced fitness of hybrids has recently been increasingly attributed to oxidative stress caused by genome mismatch^27^.

On the other hand, the male rarity has been commonly observed in natural populations of dioecious plants, without relation to hybridity^71,72^. It was particularly often observed in perennial *Rumex* species having sex chromosomes^73–78^. The phenomenon could be caused both pre- and postzygotically, most probably due to the harmful effect of partially degenerated Y chromosomes^77,79,80^. In haplophase this effect can lead to a lower proportion of male-determining pollen grains^80,81^ or certation, i.e. the lower competitiveness of these grains^79,82,83^. Disturbed sex ratios were also reported in the majority of the natural populations of two short-lived *R. hastatulus* races, but to a lesser degree than in perennial *Rumex* species^84^. The authors showed that the predominance of females depends on the population density and most probably results from prezygotic selection of competing gametophytes. The small, statistically insignificant predominance of the female sex was also observed among the progeny of T and NC plants growing under experimental conditions^16^. With regard to the obtained hybrids, they differed significantly in this respect: in TxNC the predominance of females was high, whereas in NCxT both sexes occurred in ideal balance. The difference can hardly be explained by the exceptionally strong certation in the first case and its complete lack in the latter, although it cannot be totally excluded.

It was shown that in perennial *Rumex* taxa with polymorphic sex chromosomes (XX/XY1Y2) numerical predominance of females can grow significantly in the later life-history stages^73,74,76,77,79,83^. It is generally accepted that the sex ratio biases emerging in the later life-history stages are due to the different tolerance of male and female plants to intrinsic and extrinsic stresses^38,85,86^. The different tolerance to extrinsic stresses can lead to the spatial segregation of sexes associated with microhabitat differences^87^. Unfavorable environmental conditions might cause disturbances in primary metabolism processes such as photosynthesis and respiration leading to overproduction of ROS, especially during photochemical reactions in the chloroplast or respiratory chain in mitochondria^88^. Therefore, enzymatic and non-enzymatic antioxidant systems are required to maintain intracellular ROS pools at optimal levels and mitigate potentially harmful reactions caused by enhanced oxidative load^89^. It would be interesting to investigate the response of *R. hastatulus* females and males to external stresses (e.g. drought stress) and to trace whether spatial segregation of the sexes depending on microhabitat differences occurs in the natural populations of this species.

Dowson and Geber^32^ and Juvany and Munné-Bosch^38^ discussed many examples of sex-related differences in physiological traits, but most of them concerned dioecious plants without heteromorphic sex chromosomes. The general conclusion from these studies is that the physiological differences between sexes are selection-driven and species-dependent, and that there is no general rule in this regard. Our research has shown that this also applies to the natural races of *R. hastatulus*, especially when it comes to the activity of enzymes such as SOD and CAT (Fig. 3b–d). According to the above-mentioned authors, the physiological gender dimorphisms may be especially apparent under stressful conditions, but the most common source of stress and lower survival rate of a given sex is the difference between males and females in the reproductive costs. However, it seems to play a major role in shaping of the disturbed sex ratios in populations of perennial plants, but not in annuals that end their lives after reproduction, as *R. hastatulus*.

In this paper, we demonstrated the influence of sex and hybridity—two basic factors responsible for HR—on selected components of the antioxidant system in the investigated plants. Moreover, it was shown that X-autosome asymmetries, as well as those associated with Y chromosomes and cytoplasm could modulate this system, thus affect the antioxidant response in hybrids. The T × NC hybrid exhibited the lowest M/F ratios in total activity of all analyzed antioxidant enzymes and highest DPPH scavenging activity in males. It is also the only form where the endogenous H_2_O_2_ level is higher in males than in females. It proved that the sex-related differences in the antioxidant defense system are most apparent in this hybrid and suggests that T × NC males may have difficulty with the response to hybridization stress.

Our study is the first attempt to explain the physiological aspects of HR in plants and definitely the problem requires a lot of detailed research. Regarding the analyzed parameters, we revealed important physiological differences between reciprocal hybrids, which might be connected with decreased viability of one of them. As far as genetic factors conditioning their occurrence are concerned, they seem to be similar to those in animals and related to incompatibilities between sex chromosomes (mainly X) and autosomes as well as cyto-nuclear incompatibilities. The conducted statistical analyses (PCA, Ward’s hierarchical analysis) provided quite surprising results, as they showed that physiological similarities between the examined plants to a small extent depend on genetic similarities (chromosome set), sex or race affiliation. For example, PCA analysis showed similarity of TxNC males and NCxT females (forms differing not only in sex, but also in the X and Y chromosomes), and Ward’s hierarchical analysis revealed two clusters, each of them containing an unexpected mix of genders and forms. Due to the lack of this kind of studies, it is difficult to claim whether it is a specific feature of *R. hastatulus* forms or a more common phenomenon.

## Materials and methods

### Plant material

The plants of the two original *R. hastatulus* races were obtained from seeds collected from specimens cultivated in the Department of Plant Breeding, Physiology and Seed Science, University of Agriculture in Kraków. The reciprocal hybrids of these races were cultivated from the F1 seeds obtained by free pollination in a previous experiment^16^. The used nomenclature of crosses was in line with the commonly accepted convention—the mother comes first. The chromosome complements of these plants are presented in Supporting Information (Table S3). All investigated plants were cultivated in a vegetation room under controlled conditions, i.e., at the temperature of 19 °C, under horticulture grow lights (Phytolite HPS Bloom Spectrum 400 W) and 12 h photoperiod treatment.

For measurements of PSII efficiencies two leaves were taken from five male and five female plants of each analyzed form. More plant material was used to isolate the relevant protein fractions and other biochemical analyses—an average of six leaves from each of ten individuals (of each form and sex). Measurements of chlorophyll fluorescence and leaf harvesting were performed on plants at the early beginning of flowering, to avoid internal stresses arising from reproductive costs (pollen and seed production), which could hinder interpretation of the obtained results.

The authors declare that the use of plants in the present study complies with international, national and institutional guidelines and legislation.

### Flow cytometry (FCM)

Samples for flow cytometric measurements were prepared from fresh pollen grains as previously described by Błocka-Wandas et al.^80^, using a buffer (200 mM Tris, 4 mM MgCl_2_·6H_2_O, 0.5% (v/v) Triton X-100) supplemented with propidium iodide (PI; 50 µg ml^−1^) and ribonuclease A (50 µg ml^−1^) for nuclei isolation and staining. Leaves of *Petunia hybrida* PxPc6 (2.85 pg/2C)^90^ served as an internal standard. Measurements were performed using a CyFlow SL Green (Partec GmbH, Münster, Germany) flow cytometer. Analyses were replicated four times. For each sample, 3000–5000 nuclei were analyzed. Histograms were evaluated using a FlowMax program (Partec GmbH, Münster, Germany). The coefficient of variation (CV) of *Rumex* peaks ranged from 2.95 to 4.65%. The nuclear DNA content was calculated on a histogram of fluorescence intensities using the linear relationship between the ratio of the positions of 1C peak of *Rumex* and 2C peak of *Petunia*.

### Biochemical studies

#### Isolation of soluble protein fractions and measurements of protein concentration

Plant material (1 g) was homogenized at 4 °C in 1.5 ml of 50 mM phosphate buffer with 1 mM DTT, 1% PVPP and protease inhibitor cocktail (Roche), pH 7.8 in a mortar. The homogenates were centrifuged for 5 min at 14,000 rpm. The supernatants were collected and stored at − 80 °C until further use.

Total protein concentration was determined in the supernatant according to the Bradford dye-binding method^91^ using the Bio-Rad protein assay (Bio-Rad, Hercules, CA, USA) with bovine serum albumin (BSA) as a standard.

#### Assessment of superoxide dismutase (SOD), catalase (CAT) and peroxidase (POX) activity

To determine the activities of SOD, CAT, and POX, protein fractions isolated as described above were analyzed on polyacrylamide gels (12% for SOD activity or 10% for CAT and POX activity), using the Laemmli buffer system^92^ without sodium dodecyl sulphate (SDS) at 4 °C and 180 V. Each well was loaded with the same amount of protein (5 µg). The bands corresponding to SOD isoforms were visualized on the gels using the activity staining procedure described by Beauchamp and Fridovich^93^—the gels were incubated in a staining buffer (potassium phosphate buffer, pH 7.8, containing 0.0068 g l^−1^ KH_2_PO_4_, 0.0175 g l^−1^ Na_2_HPO_4_, 0.372 g l^−1^ EDTA, 31% (v/v) Temed, 7.5 mg l^−1^ riboflavin and 0.2 g l^−1^ NBT) for 30 min. in the dark at room temperature and then exposed to white light until the SOD activity bands became visible. For identification of SOD isoforms, selective inhibitory staining was performed. H_2_O_2_ in the concentration of 5 mM was added to the staining solution in order to inhibit copper/zinc superoxide dismutase (Cu/ZnSOD) and iron superoxide dismutase (FeSOD), while 3 mM KCN was used to inhibit Cu/ZnSOD.

For visualisation of bands corresponding to CAT activity the method using the Prussian blue staining was used, as described by Woodbury et al.^94^. Before staining, the gels were incubated in 1 mM solution of H_2_O_2_.

POX activity bands were visualized using the activity-staining procedure described by Christensen et al.^95^. The gels were incubated in a staining solution (potassium phosphate buffer, pH 5, containing 0.0068 g l^−1^ KH_2_PO_4_, 0.0175 g l^−1^ Na_2_HPO_4_, 0.4285 g l^−1^ DAB in DMSO) for 20 min. Then, 25 ml of an aqueous solution of 30% hydrogen peroxide was added to the staining buffer. Directly after the appearance of the bands, the gels were transferred to water and placed at 4 °C. The gels were kept at 4 °C until densitometric analysis.

The band intensities on the gels were analyzed using the ImageJ software. Protein fractions from the studied plant material were loaded on the gel in three repetitions. Each band was measured three times. The results were presented in arbitrary units which correspond to the area under the densitometric curve.

#### Determination of endogenous hydrogen peroxide content

Endogenous concentration of hydrogen peroxide was measured using an Amplex Red Hydrogen Peroxide Assay Kit (Molecular Probes). The extract was prepared from 0.1 g of plant material which was ground in 0.5 ml of the reaction buffer (provided in the kit). Then the extract was centrifuged for 5 min at 14,000 rpm and 50 µl of the supernatant was incubated with 50 µl of the working solution containing 100 mM Amplex Red reagent and 0.2 units ml^−1^ horseradish peroxidase for 30 min at room temperature under dark conditions. H_2_O_2_ concentration was measured using a spectrofluorometer equipped with a 96-well microplate reader Synergy 2 (Bioteck) under the excitation wavelength of 530 nm and fluorescence detection at 590 nm. The standard curve in the range 0–1 µM was made using different concentrations of H_2_O_2_ provided in the kit. For each of the analyzed plant samples three measurements were done.

#### Measurement of free radicals scavenging activity with DPPH/total non-enzymatic antioxidant capacity

The total content of low molecular weight (LMW) antioxidants (free radicals scavenging activity) was measured by DPPH method according to Brand-Williams et al.^96^ with some modifications^97^, adapting the protocol to 96-well microtiter plates and to the measurement of absorbance with a microtiter plate reader. The plant material (0.1 g) was ground in a mortar in 1.5 ml of 50% methanol solution and shaken for 2 h at room temperature. The extracts were centrifuged for 20 min at 18,000×*g* and the supernatant was used for the measurements. A solution of 0.5 mM of stable free radical 1,1-diphenyl-2-picrylhydrazyl (DPPH, SIGMA) in methanol was used. The absorbance was determined after 30 min of the reaction at 37 °C at 515 nm using a reader Model 680 (Bio-Rad Laboratories, Hercules, CA, USA). The results were expressed as µmoldm^−3^ of Trolox (6-hydroxy-2,5,7,8-tetramethylchroman-2-carboxylic acid) equivalents. The standard curve in the range 0.01–0.1 µmol ml^−1^ was prepared from the stock solution of 1 mM Trolox. For one sample at least three measurements were made.

#### Measurements of PSII efficiencies—the fast chlorophyll a fluorescence kinetics

PSII efficiency was assessed on the basis of parameters calculated from OJIP test after the chlorophyll fluorescence measurement with a Handy PEA portable fluorimeter (Hansatech Instruments Ltd., King’s Lynn, Norfolk, UK), in accordance with its manual. In order to open the PSII reaction centres (RCs), the plants were adapted to darkness for 20 min. by clamping the leaf clips on upper fully developed leaves (two leaves of each analysed plant). Chlorophyll *a* fluorescence induction transients were measured when the leaves were exposed to a strong light pulse [3 mmol (photons) m^−2^ s^−1^]. The measured and calculated parameters are listed in Table S1 (in Supplementary Materials). The equations needed for calculation of the parameters were described in Strasser et al.^98^. All measurements were performed in three replicates.

### Statistical analysis

All determinations were made in three replications. Statistical analyses were done using STATISTICA 13.0 (TIBCO Software Inc., USA). The results were subjected to a one-way ANOVA, and the significance of differences between the means was determined by Tukey’s HSD test at p < 0.05. The pair-wise comparisons (e.g., of opposite sexes within a given form) were performed using Student’s *t*-test.

To compare the effect of two sexes and two types of cytoplasm on the activity of CAT, SOD, POX, the antioxidant activity (DPPH) and the amount of H_2_O_2_, an analysis of orthogonal contrasts was performed^99^. An orthogonal contrast is a weighted linear combination of treatment group means. When comparing two groups of means, e.g. females versus males plants and cytoplasm of Texas versus cytoplasm of North Carolina, the number of degrees of freedom (*df*) is reduced to one *df* so the null hypotheses were tested with Student’s *t* test (at p < 0.05).

To examine the linkage between the plant genotypes in terms of the antioxidant activity, Ward’s hierarchical clustering was applied^100^. The dendrogram was generated based on the activity of CAT, SOD, POX, the antioxidant activity (DPPH), and the amount of H_2_O_2_.

To determine the relationship between the plant genotype and the activity of CAT, SOD, POX, the antioxidant activity (DPPH) and the amount of H_2_O_2,_ the data were subjected to principal component analysis (PCA) performed in R v 3.6.1^101^ with the prcomp() function. The results were visualized with the autoplot() function from the ggfortify package v 0.4.9^102^.

## Supplementary Information

Supplementary Information.
