# Supplementary material for: Physiological aspects of sex differences and Haldane’s rule in Rumex hastatulus
Source: Sci Rep. 2022 Jul 1;12:11145. doi: 10.1038/s41598-022-15219-1 (PMC9249882; doi:10.1038/s41598-022-15219-1)
Supplement: Supplementary file 1 — Supplementary Information. [file 41598_2022_15219_MOESM1_ESM.pdf]

SUPPLEMENTARY INFORMATION

Physiological aspects of sex differences and Haldane's rule in *Rumex hastatulus*

Andrzej J. Joachimiak, Marta Libik-Konieczny, Tomasz Wójtowicz, Elwira Sliwinska, Aleksandra Grabowska-Joachimiak

FIGURES:

Figure S1. Male to female ratios (M/F) calculated for chlorophyll *a* fluorescence parameters (F0, Fm, Fv, Fv/Fm and psi_0) in analyzed *R. hastatulus* plants.

Figure S2. Visualization and identification of SOD isoforms on gel after electrophoretic separation of proteins extracted from *R. hastatulus* plants. SOD1 – MnSOD, SOD2-4 – CuZnSOD isoforms; (1) – standard solution, (2) – 5mM H_2O_2 inhibiting FeSOD and CuZnSOD, (3) – 3mM KCN inhibiting CuZnSOD.

Figure S3. Visualization of CAT activity on gel after electrophoretic separation of proteins extracted from *R. hastatulus* plants.

Figure S4. Visualization of POX isoforms on gel after electrophoretic separation of proteins extracted from *R. hastatulus* plants.

TABLES:

Table S1. Selected chlorophyll *a* fluorescence values (a.u.) in *Rumex hastatulus* plants.

Table S2. POX activity (a.u.) in analyzed *R. hastatulus* plants.

Table S3. Chromosome configuration and cytoplasm in analyzed *R. hastatulus* forms.

Figure S1. Male to female ratios (M/F) calculated for chlorophyll *a* fluorescence parameters (F0, Fm, Fv, Fv/Fm and psi_0) in analyzed *R. hastatulus* plants.

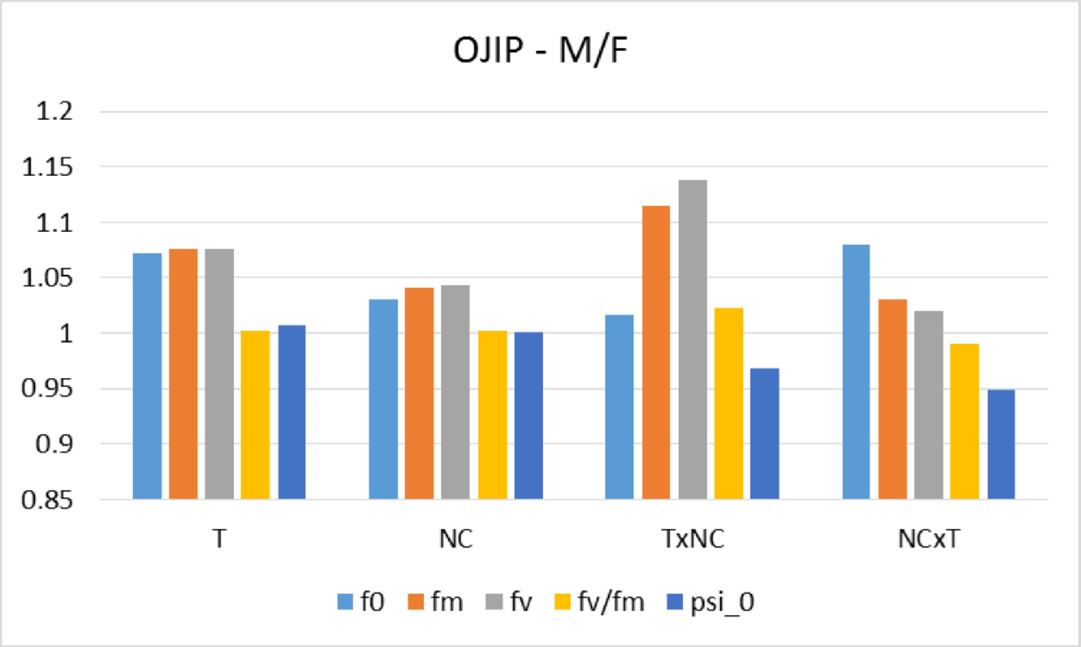

Figure S2. Visualization and identification of SOD isoforms on gel after electrophoretic separation of proteins extracted from *R. hastatulus* plants. SOD1 – MnSOD, SOD2-4 – CuZnSOD isoforms; (1) – standard solution, (2) – 5mM H_2O_2 inhibiting FeSOD and CuZnSOD, (3) – 3mM KCN inhibiting CuZnSOD.

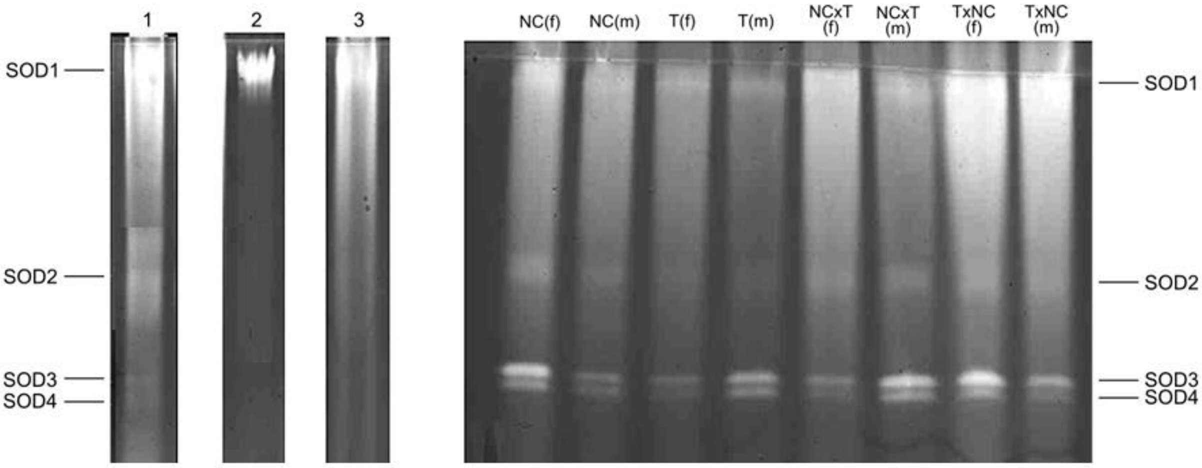

Figure S3. Visualization of CAT activity on gel after electrophoretic separation of proteins extracted from *R. hastatulus* plants. Below: full-length source gel.

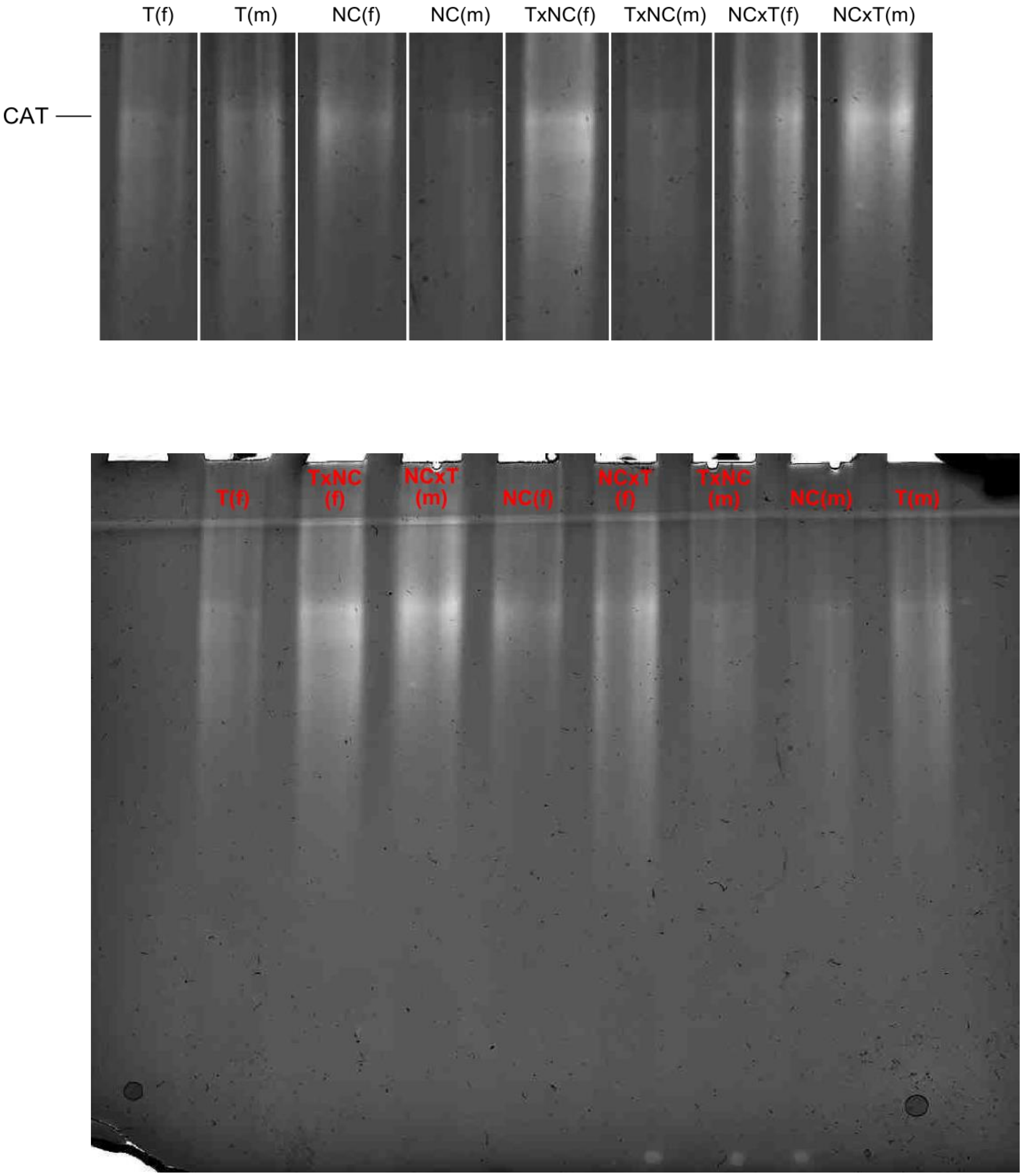

Figure S4. Visualization of POX isoforms on gel after electrophoretic separation of proteins extracted from *R. hastatulus* plants. Below: full-length POX gel.

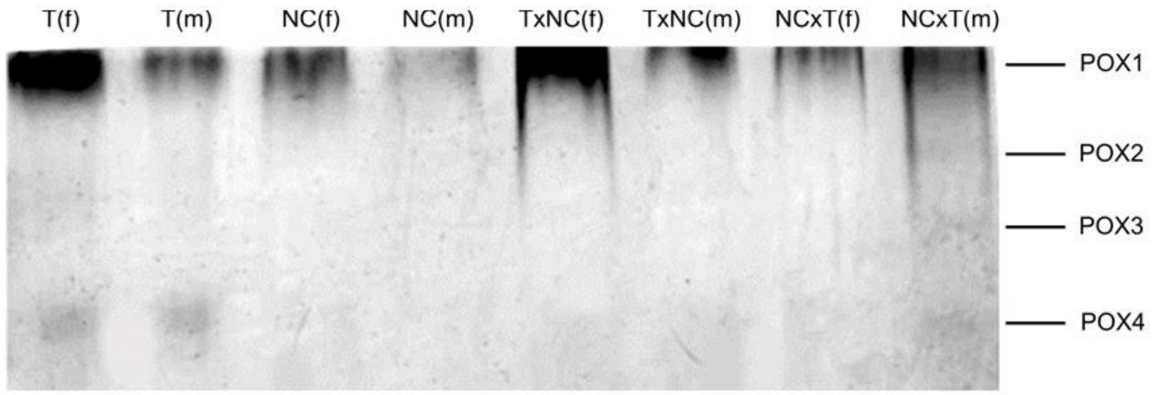

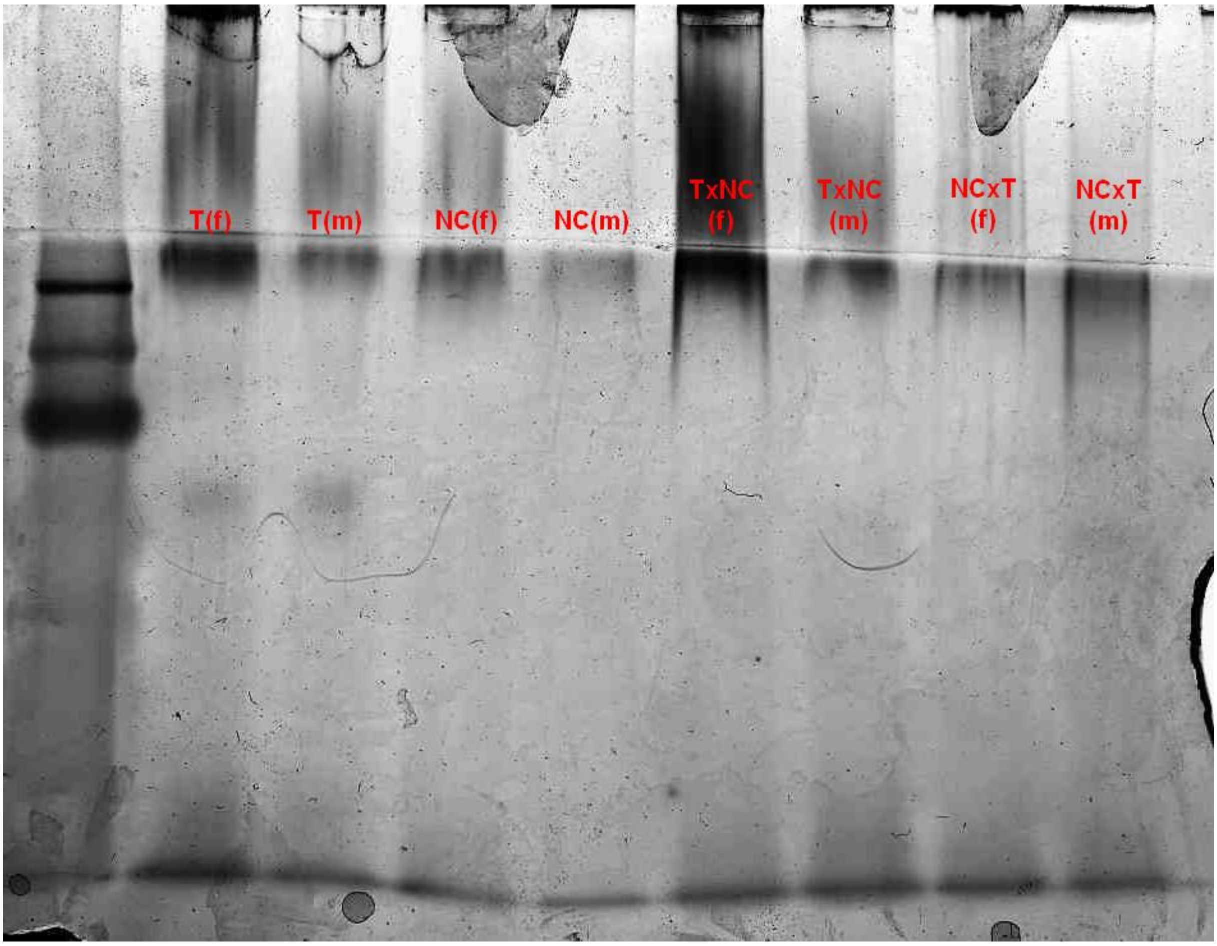

Table S1. Selected chlorophyll *a* fluorescence values (a.u.) in *Rumex hastatulus* plants.

	T(f)	T(m)	NC(f)	NC(m)	T×NC(f)	T×NC(m)	NC×T(f)	NC×T(m)
				LC3				
f0	6132.9 (485.6)	5852.0 (253.9)	5452.2 (350.7)	5449.0 (423.9)	5543.1 (750.1)	5139.9 (685.5)	5653.4 (350.5)	5457.4 (272.2)
fm	28069 (859.2)	28042.6 (1800.4)	26200.7 (726.0)	27140.0 (1787.8)	24835.3 (3239.0)	24819.1 (5845.0)	26222.1 (2132.1)	26180.9 (1231.9)
fv	21936.1 (1055.4)	22190.6 (1849.7)	20748.5 (719.1)	21691.0 (1482.8)	19292.2 (2616.4)	19679.2 (5324.7)	20568.7 (2333.6)	20723.4 (1176.0)
fv/fm	0.781 (0.019)	0.790 (0.017)	0.792 (0.013)	0.799 (0.01)	0.776 (0.016)	0.779 (0.072)	0.783 (0.026)	0.791 (0.011)
qy_max	0.780 (0.02)	0.791 (0.018)	0.791 (0.014)	0.800 (0.012)	0.776 (0.016)	0.781 (0.073)	0.783 (0.026)	0.781 (0.013)
				ojip				
f0	8586.7 (764.9)	9207.4 (417.5)	8133.8 (560.6)	8377.6 (631.8)	8033.7 (1157.4)	8166.3 (911.8)	8586.8 (764.9)	9274.7 (1082.1)
fm	45478.4 (4112.1)	48914.3 (3099.1)	45463.0 (1282.5)	47319.3 (3119.5)	40991.9 (6865.5)	45687.2 (3984.4)	45478.4 (4112.1)	46889.7 (2615.5)
fv	36891.6 (4062.8)	39706.9 (2949.6)	37329.2 (1311.0)	38941.7 (2718.0)	32958.2 (5845.6)	37520.9 (3406.3)	36891.6 (4062.8)	37615.0 (1730.8)
fv/fm	0.809 (0.023)	0.811 (0.011)	0.821 (0.012)	0.823 (0.010)	0.803 (0.015)	0.821 (0.014)	0.809 (0.023)	0.803 (0.014)
psi_0	0.527 (0.055)	0.531 (0.029)	0.564 (0.036)	0.564 (0.048)	0.543 (0.041)	0.526 (0.037)	0.527 (0.055)	0.500 (0.029)

T – Texas, NC – North Carolina, (f) – females, (m) – males, [ ] – Standard deviation

Table S2. POX activity (a.u.) in analyzed *R. hastatulus* plants.

	T(f)	T(m)	NC(f)	NC(m)	T×NC(f)	T×NC(m)	NC×T(f)	NC×T(m)
POX1	19547.36 [48.623]	14300.64 [205.618]	17141.02 [133.423]	15799.38 [726.092]	24192.54 [22.928]	14004.12 [729.927]	13425.46 [139.083]	16941.23 [619.138]
POX2	--	--	--	--	--	--	--	810.53 [12.877]
POX3	--	--	--	--	--	--	--	990.6183 [24.884]
POX4	2680.98 [29.704]	5069.754 [150.105]	--	--	--	--	--	2227.696 [73.922]

T – Texas, NC – North Carolina, (f) – females, (m) – males, [ ] – Standard deviation

Table S3. Chromosome configuration and cytoplasm in analyzed *R. hastatulus* forms.

Genotype	Chromosome configuration	Cytoplasm
T (f)	X_T•X_T•A_T•A_T	C_T
T (m)	X_T•Y_T•A_T•A_T	C_T
NC (f)	X_{NC}•X_{NC}•A_{NC}•A_{NC}	C_{NC}
NC (m)	X_{NC}•Y_{NC}•A_{NC}•A_{NC}	C_{NC}
T×NC (f)	X_T•X_{NC}•A_T•A_{NC}	C_T
T×NC (m)	X_T•Y_{NC}•A_T•A_{NC}	C_T
NC×T (f)	X_T•X_{NC}•A_T•A_{NC}	C_{NC}
NC×T (m)	X_{NC}•Y_T•A_T•A_{NC}	C_{NC}

A_T – Texas autosome set, four chromosomes;
A_{NC} – North Carolina autosome set, three chromosomes.
